# Exploring eye-movement changes as digital biomarkers and endophenotypes in subclinical eating disorders: an eye tracking study

**DOI:** 10.1186/s12888-025-06583-z

**Published:** 2025-02-14

**Authors:** Sergio Navas-León, Milagrosa Sánchez-Martín, Ana Tajadura-Jiménez, Lize De Coster, Mercedes Borda-Mas, Luis Morales

**Affiliations:** 1 Department of Psychology, Universidad Loyola Andalucía, 41704 Dos Hermanas, Spain; 2 Centro de Investigación Nebrija en Cognición (CINC), Department of Education, Universidad Nebrija, 28248 Madrid, Spain; 3 i_mBODY Lab, DEI Interactive Systems Group, Department of Computer Science and Engineering, Universidad Carlos III de Madrid, 28911 Madrid, Spain; 4 UCL Interaction Centre, University College London, London, WC1E 6BT UK; 5 Department of Applied Information Technology, University of Gothenburg, Gothenburg, Sweden; 6 Department of Personality, Assessment, and Psychological Treatment, Universidad de Sevilla, 41018 Seville, Spain

**Keywords:** Antisaccade, Eating disorders, Inhibitory control, Memory‑guided saccade, Prosaccade, Saccades, Subclinical population, Square wave jerks, Visual memory, Prevention

## Abstract

**Objective:**

Previous research has indicated that patients with Anorexia Nervosa (AN) exhibit specific eye movement changes, identified through eye tracking sensor technology. These changes have been proposed as potential digital biomarkers and endophenotypes for early diagnosis and preventive clinical interventions. This study aims to explore whether these eye movement changes are also present in individuals with subclinical eating disorder (ED) symptomatology compared to control participants.

**Method:**

The study recruited participants using convenience sampling and employed the Eating Disorder Examination Questionnaire for initial screening. The sample was subsequently divided into two groups: individuals exhibiting subclinical ED symptomatology and control participants. Both groups performed various tasks, including a fixation task, prosaccade/antisaccade task, and memory-guided task. Alongside these tasks, anxiety and premorbid intelligence were measured as potential confounding variables. The data were analyzed through means comparison and exploratory Pearson’s correlations.

**Results:**

No significant differences were found between the two groups in the three eye tracking tasks.

**Discussion:**

The findings suggest that the observed changes in previous research might be more related to the clinical state of the illness rather than a putative trait. Implications for the applicability of eye movement changes as early biomarkers and endophenotypes for EDs in subclinical populations are discussed. Further research is needed to validate these findings and understand their implications for preventive diagnostics.

**Registration:**

https://jeatdisord.biomedcentral.com/articles/10.1186/s40337-022-00573-2

## Background

Eating disorders (EDs) can be difficult to detect in routine psychiatric care [1–3]. To solve this problem, growing evidence is focusing on the identification of biomarkers and endophenotypes associated with EDs [4, 5]. Biomarkers are objectively measured indicators of biological processes, whereas endophenotypes are quantitative traits that lie in the causal pathway from genotype to phenotype [6]. While endophenotypes have a causative role, biomarkers are merely risk indicators [6]. Despite these differences, both can enhance population-based screening for identifying individuals at risk for psychopathological disorders and those who could benefit from preventive treatments [6].

In this regard, Preseller et al. [7] conducted a systematic review that identified a total of 141 studies utilizing a variety of sensors to measure aspects related to EDs. The review found that sensors can objectively assess some EDs relevant behaviors such as physical activity, food intake, and sleep patterns. Because EDs are characterized by a multifaceted symptomatology, this line of research holds promise as a complementary tool that can enhance our understanding of EDs and aid in identifying individuals at risk who may benefit from preventative interventions, contributing to more favorable therapeutic outcomes [7–11].

In this context, studies using eye tracking (i.e., sensor technology that measures eye positions and movements) have highlighted a possible attentional bias linked to disorder-related stimuli, such as food or bodies, distinctive behavioral biomarkers, suggesting an altered visual attention in individuals with EDs [6]. However, few studies have specifically addressed the role of specific eye movements patterns to disorder-unrelated stimuli [12]. In this line, Phillipou et al. [13] found that patients with Anorexia Nervosa (AN; an ED characterized by restricted food intake and an intense fear of gaining weight) showed poor performance on ocular fixations tasks, i.e., an impaired ability to maintain fixation on a single dot compared with control participants. Specifically, they showed an increased rate of saccadic intrusions named square wave jerks (SWJs; these are horizontal, involuntary, saccadic intrusions that interrupt fixation). Recently, Phillipou et al. [14] identified the state independence and heritability of this possible biomarker. According to the results, they found that patients with AN, patients’ weight- restored from the illness, and sisters of people with AN, made significantly more SWJs than healthy controls. Furthermore, the combination of SWJ rate and anxiety showed high accuracy levels to discriminate between the different groups tested (≥ 70%). Another study using prosaccade/antisaccade and memory-guided saccade tasks showed the existence of specific eye movement anomalies in patients with AN [15]. In the prosaccade task participants are instructed to fix on a central dot. Then, they have to direct their gaze toward a target dot appearing at the periphery as quickly and as accurately as possible. This task is stimulus-driven as it requires to perform a saccade (i.e., a ballistic movement of the eyes that shifts the centre of gaze) to an onset peripheral stimulus, a process which is difficult to inhibit. On the contrary, in the antisaccade task participants are instructed to look in the opposite direction of the peripherical dot. This task is goal-driven since it requires volitional processing, a voluntary saccade to the opposite direction of the stimulus. The memory-guided saccade task requires a saccade towards a remembered onset peripheral stimulus after a brief delay. Compared to control participants, patients with AN tended to show shorter saccade latencies in the prosaccade/antisaccade task and more inhibitory errors in the memory-guided saccade task [15]. In a more recent study, Phillipou et al. [16] reported that people with AN made more inhibitory errors than the weight-restored AN group, sisters of people with AN, and healthy controls. The findings demonstrate a potential state-dependent measure associated with the active clinical status of the illness [16].

Nevertheless, there is a call to focus not only on AN sample, but to also include diverse samples such as other ED subtypes [5]. According to this transdiagnostic perspective, it would be possible to find potential biomarkers or endophenotypes to identify "particular symptoms across heterogeneous illness profiles” [5]. Given the current evidence, it is not yet known whether these eye movement patterns start to manifest also in heterogeneous samples with subclinical ED symptomatology. This line of research is promising as it suggests the possible use of remote and portable eye trackers as an additional tool to screen for subclinical ED symptomatology in primary care, complementing standard assessment practices. This could contribute to enhancing prognostic and preventive treatments, an area that warrants further exploration in the field [5, 10]. Furthermore, studies using subclinical ED samples could help to shed light on whether these changes may reflect the condition itself, a trait, or a state of the illness during the active phase.

### Study objectives and hypothesis

The primary aim was to investigate whether young adult women with subclinical ED symptomatology and control participants differ in their performance on several eye movement tasks. We hypothesize that participants with subclinical ED symptomatology compared to controls would be prone to show shorter saccade latencies and more inhibitory errors in a prosaccade/antisaccade task and in a memory-guided saccade task, respectively, and an increased rate of SWJs in a fixation task. As a secondary aim, we tested the potential role of SWJ rate and anxiety to discriminate the different groups tested in case there were significant differences (as in [14]).

## Methods/design

The study was conducted in accordance with the Declaration of Helsinki and after obtaining local Ethics Committee approval (Ref: PID2019-105579RB). The experimental protocol of this study can be found in [6].

### Participants and procedure

A young adult female sample was chosen due to the higher prevalence of EDs in this population [17–19]. Thus, the sample comprised young adult women in the 18–25 age range. The sample procedure was through public advertisements and social media posts (convenience sampling). Inclusion criteria were: (a) normal (or corrected) visual acuity; (b) sex at birth: women; (c) current BMI in the normal range according to World Health Organization (WHO) (between 18.5– 24.9). Exclusion criteria: (a) self-reported lifetime history of significant brain injury, neurological condition, ocular and/or visual pathology; (b) self-reported lifetime history of an ED or other mental illness; (c) self-reported current use of psychotropic drugs (e.g., antidepressants) or intake of recreational synthetic or natural drugs; (d) incomplete data collection or eye-tracker calibration failure; (e) inability to understand Spanish; (f) out of range age. The Spanish Eating Disorder Examination Questionnaire (S-EDE-Q) [20] was administered since it is considered the gold standard for assessment of ED pathology [21]. The EDE-Q has shown good predictive as well as concurrent validity [20, 22]. Further, it has been shown to have good psychometric properties in young adults in Spain [20]. This tool served as a pre-screening tool, allowing us to split the sample into two groups of participants with subclinical ED symptomatology and control participants according to their EDE-Q score, as defined by the study. Following the suggested cut-off of 4 of Fairburn and Cooper [23], which has been widely used in previous research with both clinical [24, 25] and non-clinical samples [26–28], the responses on both EDE-Q items 22 (Weight overvaluation: “Has your weight influenced how you feel about yourself as a person?”) and 23 (Shape overvaluation: “Has your shape influenced how you feel about yourself as a person?”) were used. Participants were classified with subclinical ED symptomatology if they rated the aforementioned EDE-Q items as 4 or higher on a scale of 0 (not at all) to 6 (markedly). On the contrary, participants were classified without subclinical ED symptomatology (control group) if they rated these items below 4.^1^

Recent research supports the use of weight/shape importance as a meaningful cutoff, particularly across different ED diagnoses [29–31] and has been extensively employed in prior research [32]. This approach aligns with the transdiagnostic model of EDs [33], which posits that EDs share a common core psychopathology involving the overvaluation of body shape and weight [34, 35]. The pre-screening allowed us to form two groups of similar size: once the desired participant sample was reached for one of the groups, only participants falling into the other group defined for the study were invited to take part.

At the beginning of the experiment written informed consent was obtained from all participants. Subsequently, to identify possible confounds, we measured variables that may have affected performance in the eye movement tasks (premorbid intelligence, i.e., before the onset of the disorder, and anxiety) [15]. Only those with significant relationships between groups will be deemed potential confounders and included in the analysis.

Next, a battery of eye movement tasks presented in the same order was administered (see the Measures section for more details). After completing the study, a debriefing session was carried out. The full procedure took approximately 90 min.

### Measures

#### Psychometric measures

##### **Eating disorder symptomatology: Spanish Version of the Eating Disorder Examination Questionnaire (S-EDE-Q) **[20]

Composed by 28 7-point Likert-type response items ranging from 0 (not at all) to 6 points (markedly). Four subscales are measured, including dietary restraint, shape concerns, weight concerns, and eating concerns. The global score ranges from 0 to 6 points. Higher scores on the EDE-Q indicate higher levels of ED symptomatology. The subscales demonstrated high reliability: the Restraint subscale had an alpha of 0.829, Eating Concern was 0.827, Shape Concern was 0.943, and Weight Concern was 0.867. The global score of the S-EDE-Q exhibited exceptional reliability with an alpha of 0.961.

#### Premorbid intelligence

##### **The Word Accentuation Test **[36]

Composed by 30 low frequency Spanish words whose accents have been removed. Participants must demonstrate their knowledge of the correct accentuation of each word. The total score is the number of words correctly read (from 0 to 30). The test is administered individually and takes 2–3 min. It has demonstrated excellent psychometric properties in terms of reliability and validity in the Spanish population (del Pino et al., 2018).

#### Anxiety levels

##### **State-Trait Anxiety Inventory (STAI) **[37]

The two forms of anxiety (state and trait) are separated in the inventory, and both have their own 20 separate questions. The questionnaire is therefore composed of 40 items ranging from 0 (almost never) to 4 (almost always). The score ranges from 0 to 80 points. Higher scores on the STAI indicate higher levels of anxiety. For both forms, higher scores indicate higher levels of anxiety. The State-Trait Anxiety Inventory (STAI) showed high reliability for the state anxiety subscale (α = 0.937) but a low reliability for the trait anxiety subscale (α = 0.483).

#### Other measures

Age and highest level of education completed were collected. Body mass index (BMI) was also measured.

#### Data acquisition

Participants were invited to sit in a bright room with constant lighting and temperature in front of a 24″ LED 144 Hz monitor (Asus VG248QE; 60-Hz refresh rate) with resolution 1920 × 1080 pixels. An adjustable chin rest was used to minimize head movements and to ensure a constant distance between the participants’ eyes and the screen (90 cm). Eye movements were recorded with the Eye-Link Portable Duo (SR Research, Ontario, Canada). Both eyes were recorded at a sampling rate of 500 Hz. The device uses a dark pupil-to-cornea reflection method. A saccade velocity threshold of 30°/sec, an acceleration threshold of 8000°/s2 and a motion threshold of 0.15° was set. Before each task, an automatic randomized 9-point calibration was conducted on a black background screen and drift correction was performed throughout the task when necessary.

#### Eye movement tasks

##### **Fixation task **[13]

**Fig. 1:**
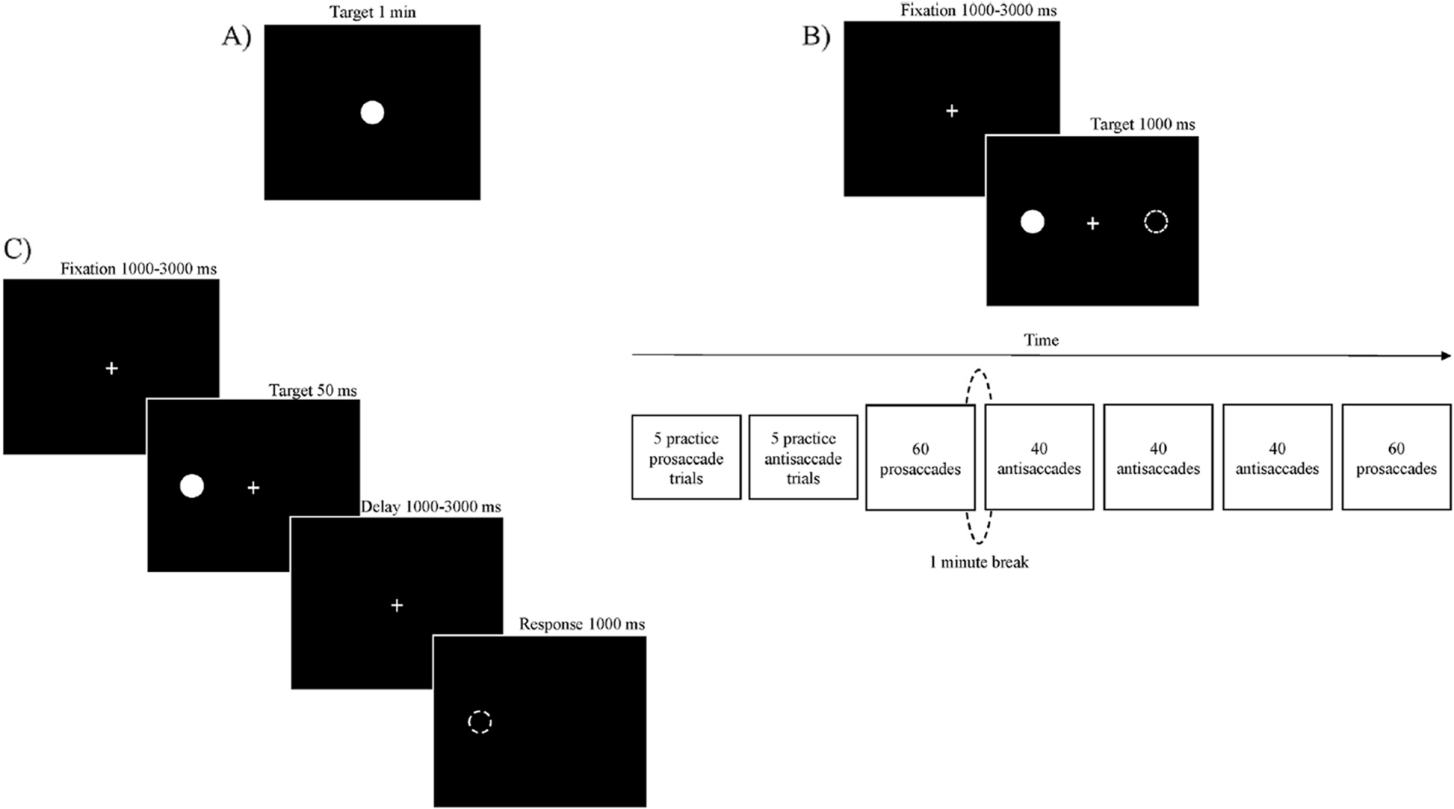
Eye-tracking task. **a** Fication task, **b** Prosaccade/ Antisaccade, **c** Memory- guided task

The participant is asked to look at a centred fixation dot (diameter of 0.5 degrees) for 1 min (see Fig. 1a). The task comprises three trials with a short resting period between them.

##### **Prosaccade/antisaccade task **[38]

Each trial begins with a centred fixation cross (random period 1000 – 3000 ms) followed by a dot (diameter of 0.5 degrees) that appears in a random location either 8º left or right of the fixation cross (1000 ms). In the prosaccade condition the participant is required to direct her gaze towards the dot as quickly as possible, while in the antisaccade condition the participant is required to direct her gaze to the opposite position of the dot (see Fig. 1b). First, participants are required to complete a practice phase consisting of five prosaccade trials and five antisaccade trials. Once practice trials are completed, a total of 240 trials are presented organized in five blocks as follows: 60 prosaccades; 40 antisaccades; 40 antisaccades; 40 antisaccades; 60 prosaccades (see Fig. 1b).

## Memory-guided saccade task [15]

The participant is asked to look at a centred fixation cross for a random period from 1000 to 3000 ms. Next, a 50 ms dot (diameter of 0.5 degrees) is presented in a random location in the periphery (5–10° left or right) while the central fixation remains for a random period in the interval 1000–3000 ms. The participant is instructed to keep her eyes on this central fixation during the entire delay and to trigger a saccade towards the memorised location of the peripheral dot as soon as the central fixation disappears. First, participants are required to complete a practice phase consisting of eight trials. Once practice trials are completed, a total of 52 trials are presented, with an equal number of target presentations for each peripheral location (see Fig. 1c).

For all the tasks: cross fixation colour, font type and size (RGB: 255,255,255; Font: Times New Roman, 28). Background screen colour (RGB: 51,51,51).

### Data analysis

For eye tracking data management, Excel 2021 (version 18.0) was used, employing scripts in Excel Macro language. Likewise, for each eye movement analysis task, a specific script was developed in the R programming language [39], taking advantage of its advanced analysis capacity and flexibility. JASP (version 0.16) was used for all statistical analyses.

#### Sample size calculation

80% power and a significance criterion of α = 0.05 were set since these are considered a convention for general use specifically in the psychology field [40, 41]. Regarding the effect size, based on previous literature on the topic [16], we choose a large effect size (d = 0.80) [40]. Thus, a priori t-test G Power v3.1.9.2 (Dusseldorf University, Germany, http://www.gpower.hhu.de/en.html) analysis yielded a minimum sample of 26 participants in each group (*N* = 52, two groups: participants with subclinical ED symptomatology vs. control participants).

#### Statistical analysis

Normality and outliers were checked through graphic tests such as QQ plots, histograms, and box plots. Group analyses was performed with Student’s t-test paired comparison (for normal distribution) or Wilcoxon tests (for non-normal distribution). We applied Benjamini–Hochberg procedure correction for multiple t-test comparisons.

In case of significant differences between study groups, we then included confounding variables in the analysis described above: premorbid intelligence and anxiety was considered a confounder for all variables based on previous literature (i.e., [13]).Across groups, exploratory Pearson’s correlation analyses were also conducted between S-EDE-Q, STAI scores, and BMI and the outcome measures associated with the eye tracking data (see Table 1, for a detailed overview of the outcome measures). Additionally, if significant differences were found between STAI scores and SWJ rate, consistent with previous literature [13, 14], a discriminant analysis would be performed on the entire sample to elucidate to what extent both measures correctly classify group membership (participants with subclinical ED symptomatology vs. control participants).

**Table 1:** Outcome measures associated with the eye‑tracking data

Task	Outcome		
Fixation	Rate of SWJs. Threshold for SWJ detection included saccade pairs (an initial saccade that moves the fovea away from the cross fixation, followed by a second saccade in the opposite direction to refoveate the fixation) within 200 ms, with amplitudes between 0.1° and 5°		
Prosaccade	Gain, latency, and peak velocity of correct saccades	Errors	-Anticipation rate: making a saccade during the fixation period, prior to the presentation of the target dot or within 80 ms of its presentation
			-Prosaccade error: saccade in the opposite direction of the peripheral stimulus
Antisaccade	Gain, latency, and peak velocity of correct saccades	Errors	-Corrected error rate: looking at the target, then looking at the correct location at the mirror image of the target
			-Latency of the correction saccade: how long it took to make a saccade in the correct direction following the incorrect saccade
			-Uncorrected error rate: looking at, instead of away from, the target
			-Anticipation rate: making a saccade during the fixation period, prior to the presentation of the target dot or within 80 ms of its presentation
Memory-guided saccade	Memory‑guided saccade Gain, latency, and peak velocity of correct saccades	Errors	-Inhibitory errors: Looking at the dot when it was presented or making a saccade before the response period
			-Directional errors: Looking in the wrong direction to where the stimulus had been presented during the response time

Practice trials were aimed to acquaint the participant with the experimental paradigms and were not statistically analyzed. In the saccade tasks, saccades smaller than 2° were not analyzed. The error rate for each participant was calculated as the proportion of erroneous trials to all valid trials. The gain of the first saccade was calculated as a ratio of the first saccade amplitude divided by the desired saccade amplitude (e.g., 8°for Prosaccade task). Latency of the first saccade was defined as the latency from appearance of the target to the start of the saccade

## Results

For the prosaccade/antisaccade task, most exclusions were due to fixation errors (4.429%), which occur when participants fail to maintain their gaze on a predetermined point. This was followed by exclusions due to exceeding amplitude thresholds (1.427%), where the eye movements exceed the defined range of motion for the task. The least number of exclusions were related to blinks (0.162%), which involve involuntary or voluntary eye closures that interfere with data collection. The total was 6.109% of trials excluded. For the memory-guided saccade task, exclusions were primarily due to exceeding amplitude thresholds (5.629%), followed by fixation errors (1.638%), and the least number of exclusions were related to blinks (0.524%). The total was 7.762% of trials excluded. After that, the percentage of trials excluded for each eye movement task was determined for each participant, using this exclusion percentage as a criterion for identifying outlier cases with boxplots. Based on this criterion, data from seven participants were excluded. On the other hand, no multivariate outliers were detected when applying the Mahalanobis distance to the psychological variables collected in the questionnaires (i.e., ED symptomatology and anxiety). The total sample size was comprised of 55 participants, with 29 in the “Control” group and 26 in the “Subclinical” group.

### Participants

Table 2 shows the descriptive statistics of the participants' sociodemographic variables. The demographic variables age and BMI evidenced no statistically significant differences between the “Control” and “Subclinical” groups, while all psychological measures used for splitting the sample showed significant differences, with significance values less than 0.001 for most comparisons. Effect sizes ranged from moderate to high. Unexpectedly, both groups differed from each other in terms of anxiety, with the control group scoring higher than the subclinical group. In order to exclude any potential effect of anxiety on our results, we compared the same variables described in the Statistical Analysis’ section above, but including anxiety as a covariate (ANCOVAs). All the analyses showed that anxiety did not have any effect in varying our results. Therefore, for the sake of simplicity, we report t-tests’ outcomes in the main text.

**Table 2:** Demographic and psychological characteristics

Variables	M (SD) Control	M (SD) Subclinical	Statistic	*p*	Effect size
Age	20.310 (1.417)	20.038 (1.428)	436.500 ^b^	.308	.158
BMI	21.285 (1.578)	21.769 (1.889)	-1.036 ^a^	.305	-.280
STAIT- State	49.714 (7.605	38.880 (9.369)	568.000 ^b^	** < .001**	.623
STAIT- Trait	40.607 (11.364)	31.120 (10.764)	530.000 ^b^	**.001**	.514
Premorbid intelligence	8.214 (3.425)	8.182 (2.612)	.037 ^a^	.971	.010
S-EDE-Q Restraint	.593 (.514)	2.462 (1.118)	-7.812 ^b^	** < .001**	-2.147
S-EDE-Q Eating concern	.345 (.362)	1.438 (1.042)	75.500 ^b^	** < .001**	-.800
S-EDE-Q Shape concern	.841 (.576)	3.288 (1.256)	-9.452 ^a^	** < .001**	-2.553
S-EDE-Q Weight concern	.434 (.421)	2.731 (1.243)	3.000 ^b^	** < .001**	-.992
S-EDE-Q Global	.553 (.310)	2.480 (.951)	-10.324 ^a^	** < .001**	-2.788

*n* 29 “Control”; *n* = 26 “Subclinical”

^a^ Student's t

^b^ Mann–Whitney U

### Fixation task

No significant differences (*p* > 0.005) were found between the “Control” group (M = 1.688; SD = 3.856) and the “Subclinical” group (M = 1.385; SD = 2.082) in terms of the number of saccadic intrusions performed per minute.

### Prosaccade/antisaccade task

In the prosaccade task (see Table 3), gain and latency between the “Control” and “Subclinical” groups showed no statistically significant differences (*p* > 0.005). Peak velocity and anticipation rate also revealed no significant differences between groups (*p* > 0.005). Prosaccade errors showed statistically significant differences, with a low effect size (*p* = 0.034). Nevertheless, after applying the False Discovery Rate (FDR) correction using the Benjamini–Hochberg procedure across the 23 comparisons, the results were non-significant. Given the criteria set by this correction method, only the most significant findings, particularly those with p-values notably less than 0.001, remain statistically significant. This adjustment process revealed that the previously noted significant difference in prosaccade errors, despite its low effect size, did not withstand the FDR adjustment.

**Table 3:** Prosaccadic task results

Variables	M (SD) Control	M (SD) Subclinical	Statistic	*p*	Effect size
Gain	.981 (.055)	.988 (.036)	-0.562 ^a^	.577	-.153
Latency	317.090 (62.814)	315.330 (91.580)	416.000 ^b^	.376	.143
Peak velocity	303.453 (45.338)	319.380 (51.128)	-1.213 ^a^	.231	-.330
Anticipation rate	.569 (.095)	.544 (.069)	1.128 ^a^	.264	.307
Prosaccade errors	.00 (.004)	.006 (.012)	292.000 ^b^	.034	-.198

*n* = 28 “Control”; *n* = 26 “Subclinical”

^a^ = Student's t

^b^ = Mann–Whitney U

In the antisaccade task (see Table 4), all comparisons between the “Control” and “Subclinical” groups, including accuracy, latency, peak velocity, anticipation rate, uncorrected error rate, corrected error rate, and latency of the corrected saccade, showed no statistically significant differences. Effect sizes were low, indicating that, indeed, differences between groups on these ocular measures are minimal.

**Table 4:** Antisaccade task results

Variables	M (SD) Control	M (SD) Subclinical	Statistic	*p*	Effect size
Gain	1.046 (.230)	1.005 (.254)	.627 ^a^	.533	.171
Latency	437.953 (100.787)	446.210 (112.458)	345.000 ^b^	.751	-.052
Peak velocity	281.549 (55.053)	280.671 (64.440)	.054 ^a^	.957	.015
Anticipation rate	.342 (.156)	.328 (.133)	376.500 ^b^	.835	.034
Uncorrected error rate	.169 (.125)	.173 (.128)	367.000 ^b^	.965	.008
Corrected error rate	.144 (.114)	.169 (.138)	327.000 ^b^	.530	-.102
Latency of the corrected saccade	540.904 (165.400)	528.069 (127.210)	410.000^b^	.434	.126

*n* = 28 “Control”; *n* = 26 “Subclinical”

^a^ = Student's t

^b^ = Mann–Whitney U

### Memory-guided saccade task

In the memory-guided saccade task (see Table 5), comparisons between the “Control” and “Subclinical" groups in terms of gain at both 5° and 10°, latency at 5° and 10°, peak velocity at 5° and 10°, as well as inhibitory errors and directional errors at 5° and 10°, revealed no statistically significant differences. Effect sizes were low for all measures collected, indicating minimal differences between groups. Notably, analysis was not performed for inhibitory errors at 5° because the variance in these data, after grouping by “Subclinical”, was equal to zero.

**Table 5:** Memory-guided saccade results

Variables	M (SD)	M (SD)	Statistic	*p*	Effect size
	**Control**	**Subclinical**			
Gain 5º	.962 (.413)	.862 (.119)	390.000^b^	.834	.034
Gain 10º	.954 (.131)	.955 (.099)	351.000 ^b^	.670	-.069
Latency 5º	705.859 (199.840)	703.792 (217.105)	387.000 ^b^	.874	.027
Latency 10º	674.987 (162.821)	658.272 (184.720)	437.000 ^b^	.319	.159
Peak velocity 5º	210.309 (42.224)	205.640 (37.169)	394.000 ^b^	.783	.045
Peak velocity 10º	278.263 (58.723)	282.047 (56.044)	-.244 ^b^	.808	-.029
Inhibitory errors 5º	.000 (.00)	.002 (.009)	- ^c^	-	-
Inhibitory errors 10º	.023 (.037)	.013 (.024)	427.000 ^b^	.316	.133
Directional errors 5º	.010 (.022)	.012 (.034)	391.500 ^b^	.726	.038
Directional errors 10º	.011 (.022)	.012 (.025)	372.00 ^b^	.919	-.013

*n* = 29 “Control”; *n* = 26 “Subclinical”

^a^ = Student's t = Student's t

^b^ = Mann–Whitney U

^c^ = Inhibitory Errors 5º analysis was not performed because the variance in the data, after clustering according to the “Group” factor, was equal to 0

### Exploratory correlational analysis

No significant correlations were found between the different measures of eye movements and the ED symptomatology variables measured by the S-EDE-Q questionnaire. The absence of significant differences precluded the discriminant analysis detailed in the protocol.

## Discussion

The goal of this research was to explore the existence of eye movement changes, previously identified in individuals with AN, within a population with subclinical ED symptomatology. Prior research has highlighted those individuals with AN exhibit an increased number of SWJs [13, 14] and a higher number of inhibitory control errors [15, 16]. Those changes have been proposed as digital biomarkers (measured by the fixation task) and endophenotypes (measured by the prosaccade/antisaccade and memory-guided tasks) [5]. Such changes in eye movement patterns may already manifest in people with subclinical ED symptomatology, which would open potential future research directions to explore the possibilities of this technique for early detection and prevention. However, until now there have been no studies making our study a novel contribution to this area of research. To achieve this, we assessed participants’ performance on a series of saccadic eye movement tasks, including fixation, prosaccade, antisaccade, and memory-guided saccade tasks. We hypothesized that participants with subclinical ED symptomatology compared to controls would be prone to show shorter saccade latencies and more inhibitory errors in a prosaccade/antisaccade task and in a memory-guided saccade task, respectively, and an increased rate of SWJs in a fixation task.

However, and contrary to our initial hypothesis [6], the findings indicate that lower-order visual processing remains well-preserved among individuals with subclinical ED symptomatology. This is somehow consistent with existing literature, which suggests that individuals with AN performed a relatively “comparable” performance to control participants on these eye movement tasks, except for the specific changes proposed above (i.e., inhibitory errors) [15, 16]. This is also in line with Beilharz et al. [42], who found no significant differences in saccadic eye movement task performance between individuals with body dysmorphic disorder and a control group, aside from a significant trend of increased anticipatory errors in the prosaccade task among the former group [42]. Phillipou et al. [16] observed, using prosaccade/antisaccade and memory-guided tasks, that individuals with AN committed only more inhibitory errors than those in the weight restored AN group, sisters of individuals with AN, and healthy controls. They suggested that these changes might be a specific endophenotype of the acute phase of the illness, reflecting underlying neurobiological impairments. Building on these findings, it would be worth investigating whether an avoidance strategy, similar to attentional biases toward food stimuli in clinical samples [43], could be observed in individuals with subclinical ED symptoms. Adapting the prosaccade task with food cues might help explore this possibility.

In addition, Phillipou et al. [13] and Phillipou et al. [14] proposed saccadic intrusions (measured by the fixation task) as a potential biomarker (i.e., a trait). According to their findings, patients with AN, those who had recovered from the illness, and sisters of people with AN exhibited significantly more intrusions than healthy controls, in contrast to our results. However, as previously discussed, it is crucial to acknowledge the limitations of our study before drawing definitive conclusions. These limitations encompass the heterogeneity of the sample with subclinical ED symptomatology and the variability in the manifestation of subclinical ED symptomatology. Considering this perspective, and as suggested by Phillipou et al. [14], the complexity of the proposed biomarker—shaped by genetic, neurobiological, and environmental factors—suggests that its presence might not manifest uniformly across diverse populations with subclinical ED symptomatology.

Given the findings, we propose that the visual processing changes identified in previous studies may be closely associated with the active clinical phase of the illness and may not start to manifest in a heterogeneous population with subclinical ED symptomatology. From a neurobiological perspective, this suggests a well-preserved functioning in brain regions associated with SWJs and voluntary saccade control. These results imply that such biomarkers (i.e., saccadic intrusions) and endophenotypes (i.e., inhibitory errors) might not be characteristic traits of heterogeneous EDs samples with subclinical symptomatology.

### Implications for preventive strategies in eating disorders

Early identification of individuals with subclinical ED symptomatology is challenging but is critical for the timely implementation of interventions and the efficacy of treatment strategies [44]. Considering the evidence supporting state-dependent eye movement changes during the active phase of the illness [16], the preservation of lower-order visual processing—indicating well-preserved cognitive functions related to inhibitory control and visual memory—could aid in differentiating individuals with subclinical ED symptomatology or in a healthy state from those with AN. This approach aligns with recent advances in the use of digital biomarkers in psychiatric research, offering new opportunities for early detection and personalized interventions through sensor technologies such as eye tracking [45]. For example, several studies emphasize the potential of utilizing web-cam based eye trackers, which remove the necessity for costly equipment, travel, and specialized personnel, making it easier to conduct regular and long-term assessments of eye movements more accessible and feasible for integration into clinical practice, especially in primary care settings [46, 47].

Despite this trend in the literature, however, there is a need to obtain more evidence regarding the presence of specific eye movement changes as potential digital markers [10, 12]. Addressing this gap might advance the development of eye tracking as a valuable diagnostic tool of saccadic changes in clinical ED settings [10]. By confirming a close relationship between these changes in subclinical populations, it could utilize leveraging artificial intelligence and machine learning to enhance the clinical validation and accuracy of the aforementioned saccadic changes (see [48]).

### Limitations, strengths and future research directions

Firstly, for certain secondary analyses, such as exploratory correlations, a post hoc power analysis indicated low statistical power (62% for detecting an effect size of ρ = 0.3 with a total sample size of 54 at an α error probability of 0.05). Conducting similar research with a larger sample size is crucial to achieve the necessary power and to obtain more definitive evidence.

Secondly, the selection of participants was based on the EDE-Q, which prioritizes concerns related to shape and weight. However, clinical observations have noted that not all EDs involve body image disturbance [49]. Some of these cases could be framed within the DSM-5R criteria for Avoidant/Restrictive Food Intake Disorder (ARFID), which shares similarities with AN regarding the limitation in the amount and/or types of food consumed [50]. In contrast to AN, ARFID does not involve distress related to body shape, weight, or size, nor fears of gaining weight [50]. This indicates that our study’s selection criteria could inadvertently exclude individuals with subclinical ED symptomatology who do not exhibit these body image concerns.

Thirdly, the protocols for prosaccade/antisaccade tasks can differ greatly among different research labs, which might lead to significant discrepancies in the results from one study to another [38, 51]. To mitigate potential issues in designing these tasks, we employed a well-established paradigm by Antoniades et al. [38] for prosaccade/antisaccade tasks. However, this methodology involves block design for each task type (i.e., trials organized in Pro-Anti-Anti-Anti-Pro blocks). In our study non-significant differences were found between the two groups. In this regard, and as emphasized by Phillipou et al. [16], future research in samples with subclinical ED symptomatology should consider increasing the cognitive demands of these tasks through the adoption of an interleaved design (i.e., with different cues indicating whether a pro-saccade or anti-saccade trial is forthcoming). That approach might enhance the sensitivity of the tasks in detecting error rates. This remains to be directly tested, however. This consideration is particularly pertinent given our sample demographic; it has been documented that individuals in their 20 s tend to reach a performance plateau in antisaccade tasks [51].

Fourthly, it is crucial to note that the presence of SWJs does not serve as a straightforward binary marker to delineate health from disease; rather, it represents a spectrum [52]. In our study, we focused on SWJ frequency (i.e., rates per minute). Contrary to our initial hypotheses, the groups did not show significant differences in SWJ frequency. This observation aligns with the limitation outlined by Zachou et al. [53]. In their review, numerous studies were identified that reported no significant differences between the clinical groups and healthy controls [53]. This highlights the necessity of establishing a clinical cutoff for SWJ frequency to improve the accuracy of diagnoses and assessments [53]. However, achieving this objective likely requires large population-based studies.

Lastly, there are some issues that may affect the generalizability of our findings. The control group exhibited higher levels of anxiety, a clinical symptom commonly associated with EDs. Although anxiety is a common issue that can affect a wide range of individuals (even those without eating disorders) particularly among young people in educational contexts, this could have influenced our results. However, note that in all the tasks we also introduced anxiety as a covariate and it did not alter our findings. How anxiety may impact eye movements changes in subclinical samples would be an intriguing avenue to explore in the future. Additionally, we recruited only females with a BMI within the range of 18.5–24.9. Although underweight, a characteristic commonly associated with AN, may not be fully addressed within this range, this approach allowed us to reduce the likelihood of medical comorbidities in the sample, thereby enhancing the generalizability of the results [54]. It is also important to note that EDs, such as those classified under Atipical Anorexia (atypAN), can occur in individuals within the normal BMI range, highlighting the heterogeneity of these conditions [50].

## Conclusions

This study aimed to investigate eye movement changes in individuals with subclinical ED symptomatology. Our findings revealed well-preserved lower-order visual processing in this population, in contrast to the changes observed in individuals with AN. These results suggest that the visual processing changes might be more closely associated with the illness’s active clinical state rather than constituting inherent traits in those at subclinical levels. Future research should address some of the limitations mentioned above to provide more conclusive evidence. Additionally, the potential of eye tracking as a non-invasive and cost-effective technology for identifying at-risk individuals before the onset of EDs merits further investigation. This aligns with the latest advancements in digital biomarkers, where integrating technologies such as eye tracking in psychiatric care can facilitate early intervention and personalized treatment strategies, ultimately enhancing patient outcomes.

## Data Availability

The data supporting the conclusions of this article will be made available by the authors on request. The scripts are available in: https://zenodo.org/records/10913309.

## Abbreviations

AN: Anorexia
BMI: Body Mass Index
EDs: Eating Disorders
STAI: State-Trait Anxiety Inventory
EDE-Q: Eating Disorder Examination Questionnaire
SWJs: Square wave jerks

## Footnotes

1 It should be noted that the original criterion for detecting EDs in the protocol published was based on a global S-EDE-Q score of ≥ 2.8, as suggested by the study of Mond et al. [21]. However, employing a cutoff score of 4 or higher for the importance of shape or weight aligns more closely with the transdiagnostic non-clinical nature of our sample. This approach is particularly pertinent given that the original validation of the 2.8 cutoff score was confined to populations diagnosed exclusively with AN and BN in primary care [22].
